# Identification of Novel Biomarkers for Pre-diabetic Diagnosis Using a Combinational Approach

**DOI:** 10.3389/fendo.2021.641336

**Published:** 2021-04-28

**Authors:** Meng-Ting Yang, Wei-Hung Chang, Tien-Fen Kuo, Ming-Yi Shen, Chu-Wen Yang, Yin-Jing Tien, Bun-Yueh Lai, Yet-Ran Chen, Yi-Cheng Chang, Wen-Chin Yang

**Affiliations:** ^1^ Agricultural Biotechnology Research Center, Academia Sinica, Taipei, Taiwan; ^2^ Graduate Institute of Biomedical Sciences, College of Medicine, China Medical University, Taichung, Taiwan; ^3^ Department of Microbiology, Soochow University, Taipei, Taiwan; ^4^ Institute for Information Industry, Taipei, Taiwan; ^5^ Graduate Institute of Medical Genomics and Proteomics, National Taiwan University, Taipei, Taiwan; ^6^ Department of Institute of Biotechnology, National Taiwan University, Taipei, Taiwan; ^7^ Institute of Pharmacology, National Yang-Ming University, Taipei, Taiwan; ^8^ Department of Aquaculture, National Taiwan Ocean University, Keelung, Taiwan; ^9^ Biotechnology Center, National Chung Hsing University, Taichung, Taiwan

**Keywords:** marker, proteomics, serum protein, type 2 diabetes, diagnosis

## Abstract

Reliable protein markers for pre-diabetes in humans are not clinically available. In order to identify novel and reliable protein markers for pre-diabetes in humans, healthy volunteers and patients diagnosed with pre-diabetes and stroke were recruited for blood collection. Blood samples were collected from healthy and pre-diabetic subjects 12 h after fasting. BMI was calculated from body weight and height. Fasting blood glucose (FBG), glycated hemoglobin (Hb_A1C_), triglyceride (TG), total cholesterol, high-density lipoprotein, low-density lipoprotein (LDL), insulin and albumin were assayed by automated clinical laboratory methods. We used a quantitative proteomics approach to identify 1074 proteins from the sera of pre-diabetic and healthy subjects. Among them, 500 proteins were then selected using Mascot analysis scores. Further, 70 out of 500 proteins were selected *via* volcano plot analysis according to their statistical significance and average relative protein ratio. Eventually, 7 serum proteins were singled out as candidate markers for pre-diabetes due to their diabetic relevance and statistical significance. Immunoblotting data demonstrated that laminin subunit alpha 2 (LAMA2), mixed-lineage leukemia 4 (MLL4), and plexin domain containing 2 (PLXDC2) were expressed in pre-diabetic patients but not healthy volunteers. Receiver operating characteristic curve analysis indicated that the combination of the three proteins has greater diagnostic efficacy than any individual protein. Thus, LAMA2, MLL4 and PLXDC2 are novel and reliable serum protein markers for pre-diabetic diagnosis in humans.

## Introduction

Diabetes is a metabolic disease characterized by hyperglycemia and defective carbohydrate metabolism resulting from insulin resistance/deficiency with β-cell dysfunction (1). In 2019, the International Diabetes Federation estimated that 9% of adults worldwide, 463 million people, lived with diabetes (2). Moreover, this disease caused 5 million deaths in the same year (2). Total health expenditure for diabetes and its complications was estimated to be 760 billion US dollars in 2019 (2). Of note, type 2 diabetes (T2D) accounted for over 90% of all diagnosed cases (2).

Despite advanced diagnosis and significant improvement in therapies, T2D remains an incurable disease. Accumulating evidence shows that early prevention and intervention can significantly reduce the incidence of T2D (3, 4) and its complications (5), suggesting that a strategy of prevention may well be better than a cure for diabetes. Indeed, diet control, exercise, and bariatric surgery could prevent T2D in high-risk subjects (4, 6, 7). Consistently, prophylaxis with metformin also decreased the incidence of T2D (3). Therefore, identification of subjects at high risk for T2D before its clinical onset is the key to preventing this disease. To date, great efforts have been made to identify genetic and protein markers for future T2D (8–12). Although genetic markers have high reliability, they are not satisfactory because they show up across the whole lifespan of patients with T2D, not specifically at the onset of the disease or early in its progression, and sometimes have only modest sensitivity and specificity (13). On the other hand, protein markers have high sensitivity and specificity for T2D because they reflect the progression of the disease systemically and dynamically (13). Furthermore, proteins are tightly regulated by cellular stimulation; whereas genes are not (12). Thus, protein markers are practical and have potential for diabetic diagnosis.

T2D is an inflammatory disease primarily caused by obesity, insufficient physical activity and unhealthy life style (14). The role of inflammation in prevention and control of T2D is intriguing and not completely understood (14). Serum proteins related to inflammation have potential for use as diabetic markers because blood sampling is minimally invasive and feasible for any patient posing minimal risk, and serum proteins can reflect T2D pathophysiology. For example, cluster of differentiation 14 (CD14), a monocyte differentiation antigen, has been reported to modulate inflammation-driven insulin resistance and has been identified as an inflammatory marker in women with diabetes and impaired glucose tolerance (15, 16). Serum amyloid (SA) proteins such as SAA2 and SAA4 are serum proteins that are upregulated during inflammation. SAA2 was reported to be increased in the plasma of people with obesity and insulin resistance. Similarly, SAA2 was also shown to be a marker of insulin resistance in mice (17). *SAA2* gene is adjacent to *SAA4* gene on chromosome 11. A read-through transcription was found to occur naturally, leading to the generation of the SAA2-SAA4 fusion protein (Gene ID: 100528017). Additionally, some inflammation-irrelevant proteins are also used as diabetic markers. For instance, cluster of differentiation 99 (CD99) and clusterin (CLU) have been patented as diabetic markers (3, 10). CD99 was patented as a marker for insulin resistance (WO2006063733A1). Two isoforms of CD99 have been reported. A long form, CD99wt, contains 185 amino acids (32 KD) whereas an alternative splicing isoform, CD99sh, contains 161 amino acids (28 KD) (18). CD99wt is expressed on thymocytes, pancreatic islet cells, peripheral T cells, endothelial cells, and hematopoietic stem cells (19, 20). Moreover, it plays an essential role in many biological functions, including cell adhesion, migration, apoptosis, death, differentiation, intracellular membrane protein trafficking, endocytosis, and exocytosis (18, 20). CLU has been reported to be a serum marker for T2D (US8673644B2). CLU is expressed ubiquitously in the cytosol of cells, plasma, and body fluids (21). It is a multifunctional protein involved in the regulation of proliferation, differentiation, and survival of cells including epithelial cells, smooth muscle cells and synoviocytes (22). CLU has several isoforms with molecular weights of 37 KD (23), 49 KD (21), and 60 KD to 75 KD (21). Some isoforms might have different glycosylation sites (21). Although biomarkers for diabetic diagnosis may not be related to diabetes, we selected the serum proteins associated with inflammation and/or diabetes for further confirmation. In our study, we identified 7 proteins as potential markers. Four of them, CD14, SAA2, CD99 and CLU, were published diabetic markers. The other three are mixed-lineage leukemia 4 (MLL4), laminin subunit alpha 2 (LAMA2) and plexin domain containing 2 (PLXDC2) and are unknown for diabetic diagnosis. MLL4 was reported to interact with the transcription factors to regulate islet β-cell function (24). LAMA2 mutation was shown to cause merosin-deficient congenital muscular dystrophy (25), and PLXDC2 is known to regulate differentiation and proliferation during the development of the nervous system (26). These three proteins may be related to diabetes and it complications.

Proteomics approaches are emerging as a straightforward strategy to identify biomarkers for disease diagnosis. Mass spectrometry (MS)-based quantitative proteomics analysis is a highly sensitive technique to measure biomolecules at the femtomolar level as exemplified in cancer and cardiovascular diseases (27, 28). The isobaric tags for relative and absolute quantitation (iTRAQ) method can further label proteins in an isobaric manner and assist in simultaneously quantifying the amount of proteins from different sources. Over recent years, many publications have used proteomics approaches to identify the markers for T2D or its complications in human patients and/or diabetic mice (29–38). However, markers for pre-diabetes or before the onset of diabetes have not been identified.

In this study, we combined iTRAQ with MS techniques to globally characterize serum proteins of human and mouse origin at the pre-diabetic stage. Statistical significance, expression ratio, functional analysis, and molecular novelty reduced the number of human serum proteins from 1074 to 7. Ingenuity pathway analysis (IPA) was used to predict the likely interaction network and pathways of these 7 proteins. Furthermore, immunoblotting analysis and receiver operating characteristic (ROC) curve analysis were used to assess the serum expression level and diagnostic power, respectively, of these proteins in healthy and pre-diabetic subjects.

## Materials and Methods

### Chemicals and Reagents

Acetonitrile (ACN) was purchased from Merck (Hesse, Germany). Tris(2-carboxyethyl)phosphine (TCEP), methyl methanethiosulfonate (MMTS), triethylamonium bicarbonate (TEAB), trifluoroacetic acid (TFA) and other reagents were purchased from Thermo Fisher Scientific (MA, USA). Potassium dihydrogen phosphate (KH_2_PO_4_) and potassium chloride (KCl) were obtained from Sigma-Aldrich (Hesse, Germany).

### Human Serum Sample Collection

All participant signed informed content approved by the IRB/REC of the China Medical University hospital (IRB No. CMUH105REC2001). Healthy volunteers (n=20) and patients diagnosed with pre-diabetes (n=19) and stroke (n=10) were recruited at the China Medical University hospital for blood collection. There were 23 females and 26 males all of whom were Taiwanese Han. The age range was from 23 to 69 years old. Blood samples were collected from healthy and pre-diabetic subjects 12 h post fasting. The serum samples were separated from whole blood, aliquoted to avoid repeat freeze thaw cycles and then stored at -80°C. Body mass index (BMI) was calculated from body weight and height. Fasting blood glucose (FBG), glycated hemoglobin (Hb_A1C_), triglyceride (TG), total cholesterol, high-density lipoprotein, low-density lipoprotein (LDL), insulin and albumin were assayed by automated clinical laboratory methods.

### Depletion of Abundant Proteins

In order to augment the detection and identification of low-abundance proteins, the ProteoPrep immunoaffinity Albumin and IgG Depletion Kit from Sigma-Aldrich was used to evaluate the efficiency of high abundance protein depletion from serum samples using the manufacturer’s protocol. The protein concentration was calculated using the BCA protein assay kit from Thermo Fisher Scientific.

### Protein Digestion and iTRAQ Labeling

An equal amount of total protein (100 μg) per depleted sample was diluted with 0.5 M TEAB, reduced with 5 mM TCEP at 60°C for 1 h, alkylated using 10 mM MMTS at room temperature for 10 minutes and then digested with 10 μg trypsin (Promega, WI, USA) at 37°C for 16 h. Subsequently, each sample from humans was labeled with a different iTRAQ tag (Applied Biosystems, MA, USA) as follows: iTRAQ-114 was used to label the pooled serum of 3 healthy volunteers and iTRAQ-115, 116, 117 were used to label the serum of pre-diabetic subjects, respectively. The four samples from humans were combined, dried by SpeedVac, dissolved in 200 μl of 5% ACN solution containing 0.5% TFA, and desalted using a C18 spin column (Thermo Fisher Scientific). After drying with SpeedVac again, each sample was dissolved in 400 μl of 25% ACN solution containing formic acid (FA).

### Fractionation With Strong Cation Exchange Chromatography

The iTRAQ labeled samples were fractionated separately *via* strong cation exchange chromatography using polysulfoethyl A column [2.1 x 200 mm, 5 μm particle size, (PolyLC, MD, USA)] with a flow rate of 0.3 ml/min and mobile phase (A) 10 mM KH_2_PO_4_ in 25% ACN (pH 3) and (B) 1 M KCL and 10 mM KH_2_PO_4_ in 25% ACN (pH 3). The gradient of fractionation was set as follows: 0% B for 5 minutes, 0–20% B for 55 minutes, 20–60% B for 10 minutes, 60% B for 10 minutes and 60-0% B for 20 minutes. The fractions were collected and dried with SpeedVac.

### LC-MS/MS Analysis and iTRAQ Data Analysis

The dried fractions were dissolved in 200 μl of 5% ACN solution containing 0.5% TFA and desalted using a C18 spin column. Following drying with SpeedVac, the pellet was dissolved in 40 μl of 5% ACN solution containing 0.1% FA for LC-MS/MS analysis. Q Exactive mass spectrometer (Thermo Fisher Scientific) coupled with HCD fragmentation mode was used to generate MS and MS/MS spectra. Ultimate 3000 RSLC system (Thermo Fisher Scientific) equipped with a C18 column (Acclaim PepMap RSLC, 75 μm x 150 mm, 2 μm, 100 Å) was used for LC separation with a flow rate of 0.25 μl/min and the mobile phase (A) 0.1% FA and (B) 95% ACN/0.1% FA. The gradient of analysis was as follows: 1% B for 5 minutes, 1-25% B for 25 minutes, 25–60% B for 15 minutes, 60-80% B for 5 minutes, 80% B for 10 minutes, 80-99% B for 5 minutes and 99% B for 5 minutes. Relative protein ratio and peptide identification were processed by Proteom Discover 1.4 for Mascot database search. All tandem mass spectra were searched for species of *Homo sapiens* and *Mus musculus* against the International Protein Index human and mouse V 3.87 database. The mass spectrometry proteomics data have been deposited to the ProteomeXchange Consortium *via* the PRIDE partner repository with the dataset identifier PXD017472 (http://proteomecentral.proteomexchange.org/cgi/GetDataset).

### Protein Signaling Pathways and Functional Analysis

Functions and signaling pathways of serum proteins with differential expression between the healthy and pre-diabetic humans were analyzed by IPA (Ingenuity Systems, at http://www.ingenuity.com) and PubMed (at https://www.ncbi.nlm.nih.gov/pubmed).

### Immunoblotting Analysis

Serum samples were collected from healthy and pre-diabetic human subjects and then lysed by RIPA lysis buffer. Total protein (20 μg) of each serum from control and pre-diabetic human subjects and mice was resolved by 6% and 10% sodium dodecyl sulfate polyacrylamide gel electrophoresis, transferred onto nitrocellulose membrane (Schleicher and Schuell, NH, USA), immunoblotted with the antibodies against LAMA2 (1:500, LifeSpan BioSciences, WA, USA), MLL4 (1:500, Abcam, Cambs., UK), PLXDC2 (1:500, Novus Biologicals, CO, USA), CD99 (1:200, Invitrogen, MA, USA), CLU (1:500, Novus Biologicals), CD14 (1:1000, Abcam), SAA2 (1:1000, Abcam) and horseradish peroxidase (HRP)-conjugated goat and rabbit anti-mouse IgG as secondary antibody. The membranes were detected using FluorChem HD2 system (Bio-Techne, MN, USA) after developing with enhanced chemiluminescence (ECL) substrate (EMD Millipore, MA, USA). As published previously (39, 40), briefly, the accuracy, sensitivity and specificity were analyzed according to number of controls or patients divided by the total number of subjects. ROC curves were used to represent the sensitivity and specificity to (pre-)diabetic diagnosis at different cut-off values. The cut-off values of biomarkers for diagnosis were based on the best Youden index. A *p* value less than 0.05 was considered statistically significant.

### Enzyme-Linked Immunosorbent Assay Analysis

Human LAMA2 ELISA kits were purchased from Cusabio (CSB EL012726HU, TX, US). One hundred microliters of sera from healthy volunteers and patients with pre-diabetes were added to wells of a 96-well microplate. Following incubation for 2 h at 25°C, the plate was incubated with biotin-antibody and peroxidase-conjugated avidin. After extensive washing, the plate was incubated with TMB and measured for absorbance at 450 nm using an ELISA reader.

### Statistical Analysis

The data are expressed as mean ± standard deviation of the mean. Student’s t-test was used to compare the difference between healthy volunteers and patients. A *p* value less than 0.05 was considered statistically significant. False discovery rate (FDR) was used to adjust proteomic *p* value for multiple comparisons.

## Results

### Proteins Differentially Expressed in Healthy and Pre-diabetic Sera of Human and Mouse Origin

**Figure 1:**
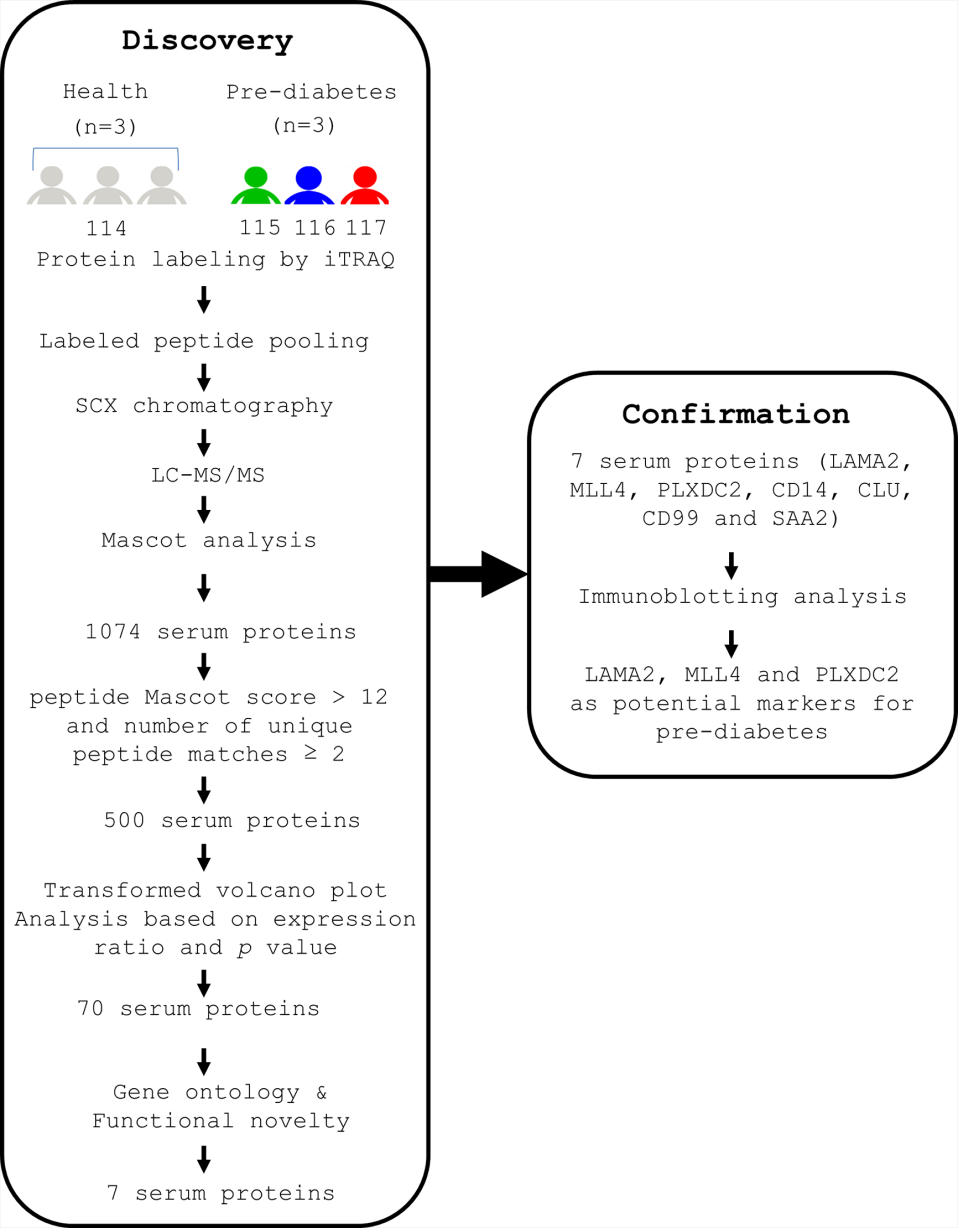
A flow chart indicating the experimental designs for identification of serum proteins of human origin, followed by confirmation of their presence in human sera. Serum samples were collected from 3 healthy volunteers and 3 pre-diabetic patients after fasting for 12 h. The serum from the 3 healthy subjects was pooled together and labeled with iTRAQ 114. The serum from 3 pre-diabetic subjects was labeled with iTRAQ 115, 116 and 117, respectively. In the first step (Discovery stage), the iTRAQ-labeled sera were pooled, depleted of serum abundant proteins, and subjected to MS analysis. As a result, 1074 serum proteins were identified. Seven serum proteins were selected from 1074 proteins by using average relative protein ratio, P value and novelty. In the second step (Confirmation stage), immunoblotting analysis showed that 3 out of the 7 serum proteins are promising markers for (pre-)diabetes.

To characterize novel and reliable markers for pre-diabetes, we first used a combination of iTRAQ and MS techniques to analyze the serum proteins of healthy (diabetes-free) subjects and pre-diabetics as described (Discovery, **Figure 1**). BMI and serum biochemistry of the human subjects were analyzed (**Supplementary Table 1**) (41). We found that age, FBG, Hb_A1C_, fasting insulin, and albumin were significantly different (*p* ≤ 0.05) between the healthy and pre-diabetic groups (**Supplementary Table 1**) (41). Serum samples from both groups were collected and their abundant proteins were then depleted, followed by trypsin digestion. Next, three serum samples from healthy subjects were pooled together to minimize individual variability and then labeled with iTRAQ 114. Three serum samples from pre-diabetic patients were labeled with iTRAQ 115, 116 and 117, respectively. Finally, four iTRAQ samples were mixed up and analyzed by LC-MS/MS (**Figure 1**). The identity of the serum proteins from healthy and pre-diabetic subjects was confirmed using the Mascot software (**Figure 1**). A total of 1074 human serum proteins were identified (ProteomeXchange, PXD017472). Five hundred proteins were selected based on the following criteria; peptide Mascot score > 12 and number of unique peptide matches ≥ 2 (**Figure 1**).

**Figure 2:**
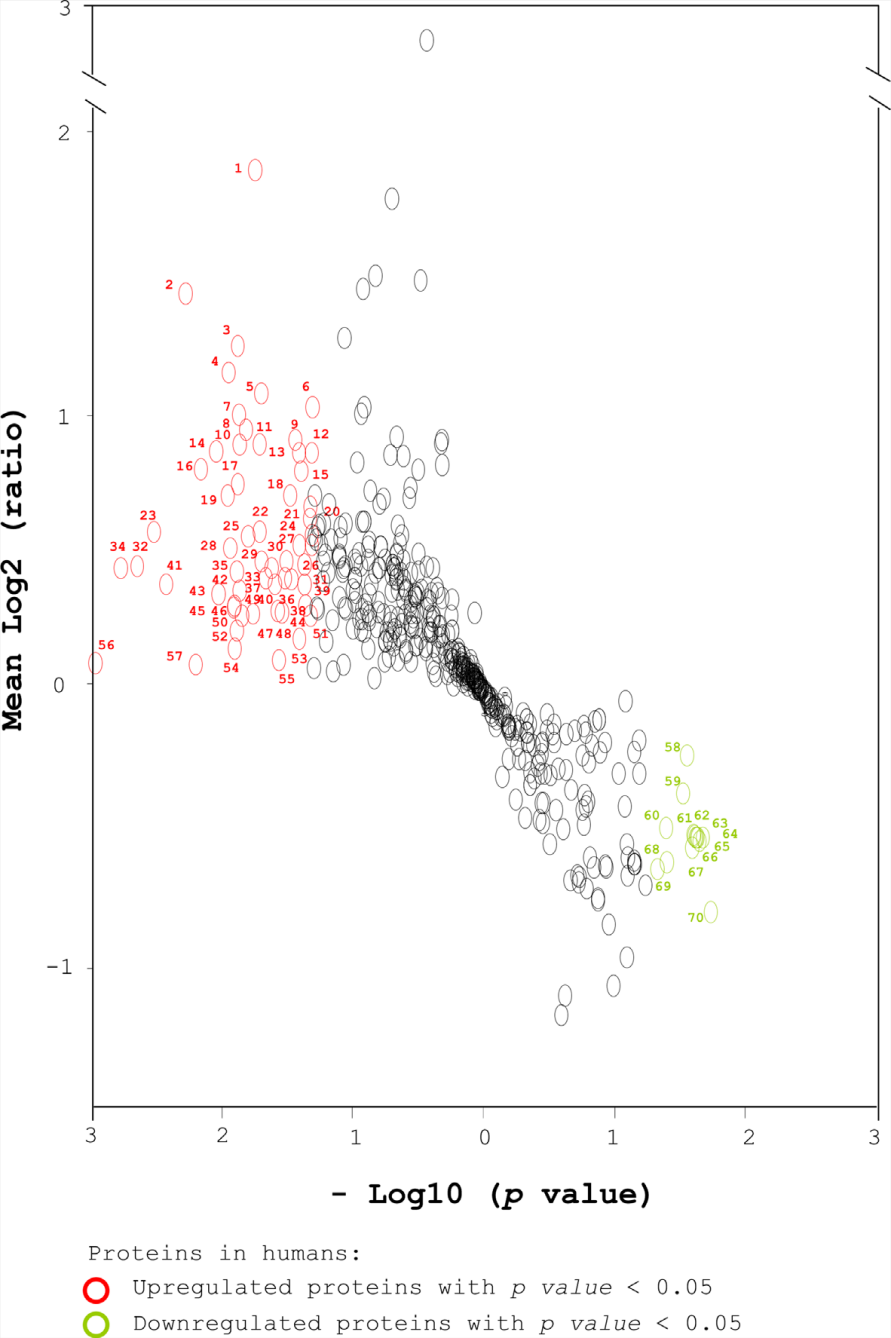
Transformed volcano plot analysis of the selected proteins from human sera. Total proteins from human sera were labeled with iTRAQ tags, followed by MS analysis and Mascot identification. A total of 1074 human serum proteins were identified (ProteomeXchange, PXD017472). Five hundred proteins were selected based on a peptide Mascot score > 12 and number of unique peptide matches ≥ 2 (**Figure 1**). A transformed volcano plot was used to analyze log2 (ratio of the level of one serum protein in a pre-diabetic patient to its average level in healthy subjects). The serum proteins detected in humans are labeled with circles. Upregulated and downregulated proteins with *p* < 0.05 (*) are labeled in red and green respectively. The proteins with *p* < 0.01 (**) that were associated with diabetes, diabetic complications and obesity were selected for further analysis using IPA. Student’s t-test was used to compare the differences between healthy and pre-diabetic humans.

To further evaluate the potential of serum proteins as pre-diabetic markers, volcano plot analysis of 500 human serum proteins was performed based on both the average relative protein ratio and the *p* value (**Figure 2**). The transformed volcano plot data indicated that among the 500 proteins, 70 proteins with *p* < 0.05 could be candidate markers for pre-diabetes (**Figure 2**) and need to be verified. In parallel, we followed the same approach to characterize the serum proteins of diabetes-free and pre-diabetic db/db mice in an attempt to compare the markers in humans and mice (**Supplementary Figure 1**) (41). Body weight (BW) and serum biochemistry are shown (**Supplementary Table 2**) (41). Healthy and pre-diabetic mice were grouped based on their FBG. Accordingly, we found that BW, FBG, Hb_A1C_, TRIG, LDL, and fasting insulin were significantly different in both groups (**Supplementary Table 2**) (41). Serum samples were collected from mice. After their abundant proteins were depleted, the remaining proteins were digested with trypsin. Similarly, sera of 3 healthy mice were pooled and labeled with iTRAQ 114m. Sera of 3 pre-diabetic mice were labeled with iTRAQ 115m, 116m and 117m, respectively. A pool of four iTRAQ samples were analyzed by LC-MS/MS and their identity was ascertained using Mascot analysis (**Supplementary Figure 1**) (41). Finally, 995 serum proteins were chosen from mice (ProteomeXchange, PXD017472) with the same criteria as in the human cohorts (**Supplementary Figure 1**) (41).

### Gene Ontology and Pathway Analysis of the Selected Serum Proteins

To gain information about the biological function of the 70 human proteins (**Figure 2**) that had been selected using statistical significance (*p* < 0.05) and average relative protein ratio, these proteins was analyzed by gene ontology and by searching PubMed (**Supplementary Figure 2**) (41). The proteins could be classified into 6 functional categories; related to diabetes, diabetic complications, obesity, inflammatory immunity, coagulation and others (**Supplementary Figure 2**) (41).

Next, we narrowed down the number of candidate markers by picking up proteins based on higher *p* values (< 0.01) and functions associated to diabetes, diabetic complications and obesity. Seven proteins, LAMA2, MLL4, PLXDC2, CD14, CLU, CD99 and SAA2/4 stood out when these stringent selection criteria were used (**Figure 2** and **Supplementary Table 4**). This discovery strategy for potential markers of (pre-)diabetes uncovered several markers already known to be associated with diabetes. For example, CD99 (3, 10) and CLU (3, 10) have been patented as diabetic markers. SAA2 (17) has been reported to increase in the plasma of obese and insulin resistant humans and was a marker of insulin resistance in mice. CD14 (15, 16) has been reported to modulate inflammation-driven insulin resistance and was identified as an inflammatory marker in women with diabetes and impaired glucose tolerance. CD99, CLU, CD14, and SAA2 have been reported to be associated with diabetes from human serum proteins. The data suggest the feasibility of the identification strategy using a combination of quantitative proteomics, statistical analysis, and functional analysis to seek protein markers for diabetes. However, whether LAMA2, MLL4 and PLXDC2 can be applied as (pre-)diabetic markers remains unclear.

**Figure 3:**
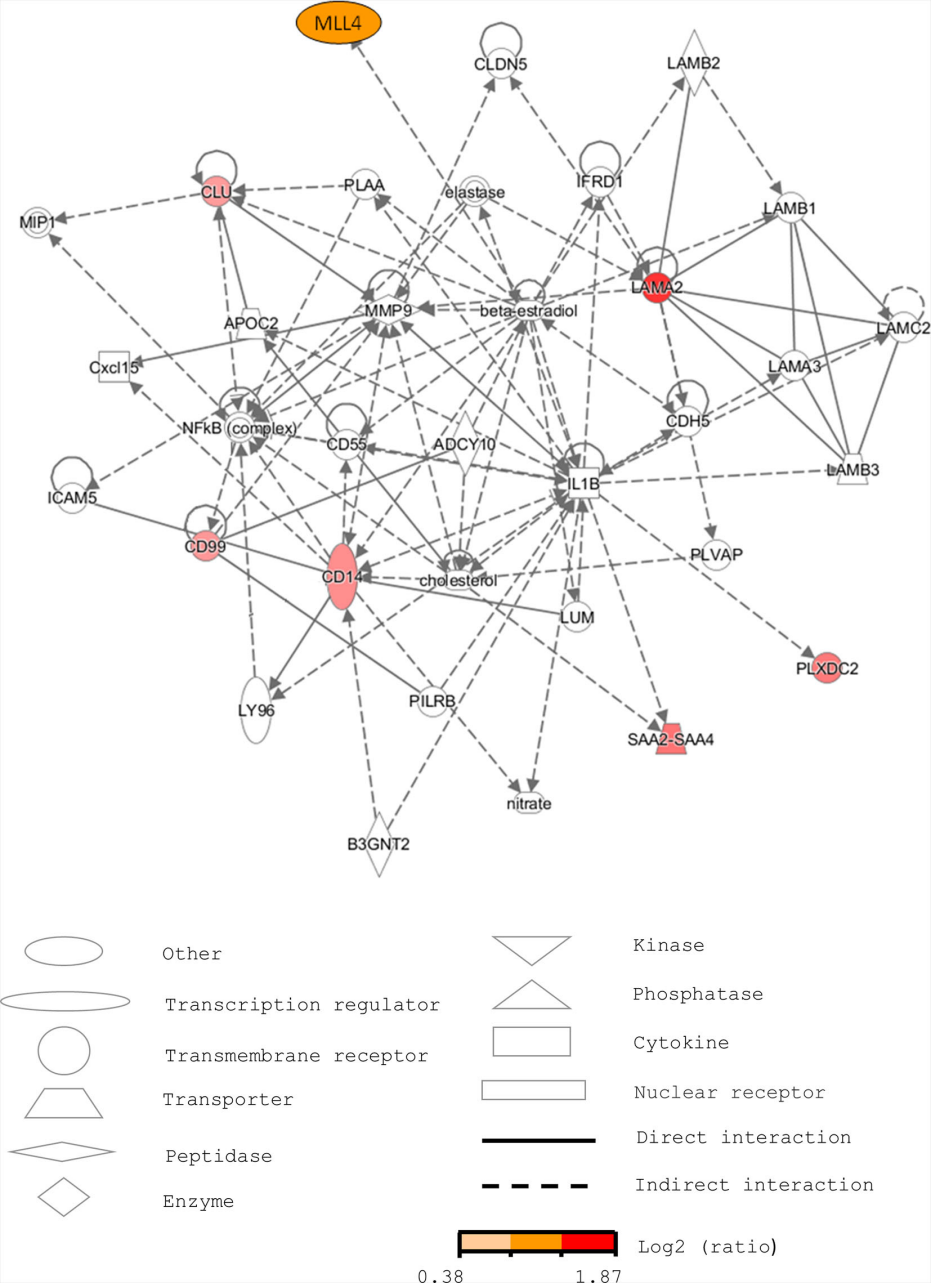
Pathway analysis of the selected serum proteins from human sera using IPA. The network is related to connective tissue disorders, dermatological diseases and conditions, and developmental disorders. The markers increased in pre-diabetic patients are labeled in red.

To better understand the biological meaning of the changes in the levels of these proteins before and during T2D, web-based IPA (42) and PubMed database searches were used to predict the protein signaling pathways. IPA generated a network of a total of 35 proteins related to connective tissue disorders, dermatological diseases and conditions, and developmental disorders (**Figure 3**). Of course, the putative signaling pathways need to be ascertained by further experiments.

### Confirmation of the Selected Proteins as Potential Markers for Pre-diabetes

**Figure 4:**
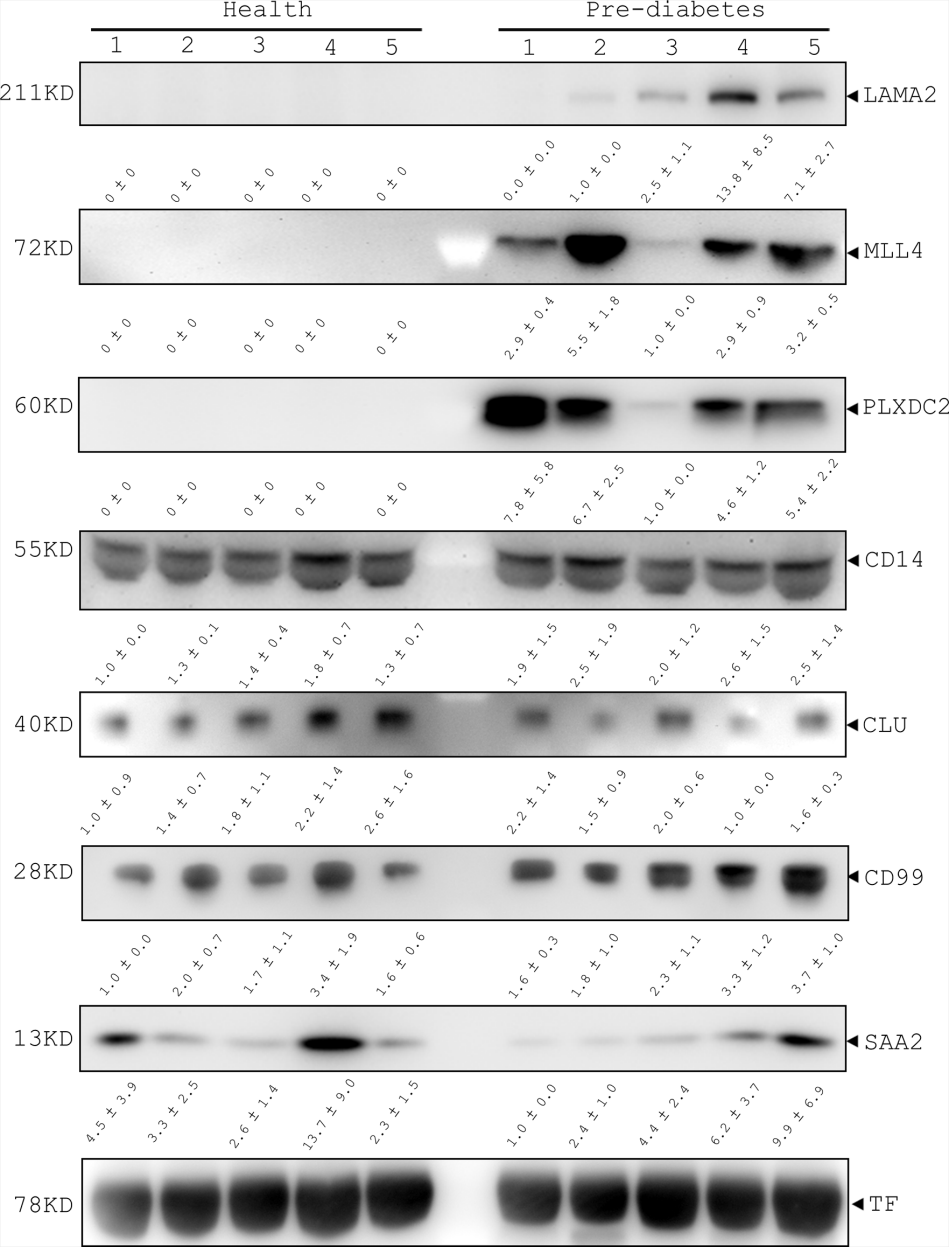
Immunoblotting analysis of LAMA2, MLL4, PLXDC2, CD14, CLU, CD99, and SAA2 in healthy and pre-diabetic sera of human origin. Serum samples of healthy and pre-diabetic subjects were collected and then lysed with lysis buffer. After centrifugation, total lysates were prepared for immunoblotting analysis with the antibodies as indicated. Transferrin (TF) was used as an internal control of human sera.

To confirm the feasibility of using the 7 human serum proteins (LAMA2, MLL4, PLXDC2, CD14, CLU, CD99 and SAA2) as pre-diabetic markers, we conducted immunoblotting analysis. The immunoblotting data showed that LAMA2, MLL4 and PLXDC2 were undetectable in the sera of 5 healthy volunteers and their expression was highly regulated in pre-diabetic patients although their levels varied (LAMA2, MLL4 and PLXDC2, **Figure 4**). Additionally, we examined the expression level of CD14, CLU, CD99, and SAA2 in the sera of healthy subjects and pre-diabetic patients. No significant difference in the serum level of CLU, CD99, CD14 and SAA2 was observed between the two human cohorts (**Figure 4**). Moreover, we tested the specificity of the 7 selected proteins in the sera of stroke patients. The immunoblotting showed that LAMA2, MLL4 PLXDC2 and CD99 were not detected in the sera of 10 stroke patients (**Supplementary Figure 3**) (41). However, we found that CD14, CLU and SAA2 were expressed in the sera of stroke patients at various levels (**Supplementary Figure 3**) (41).

**Figure 5:**
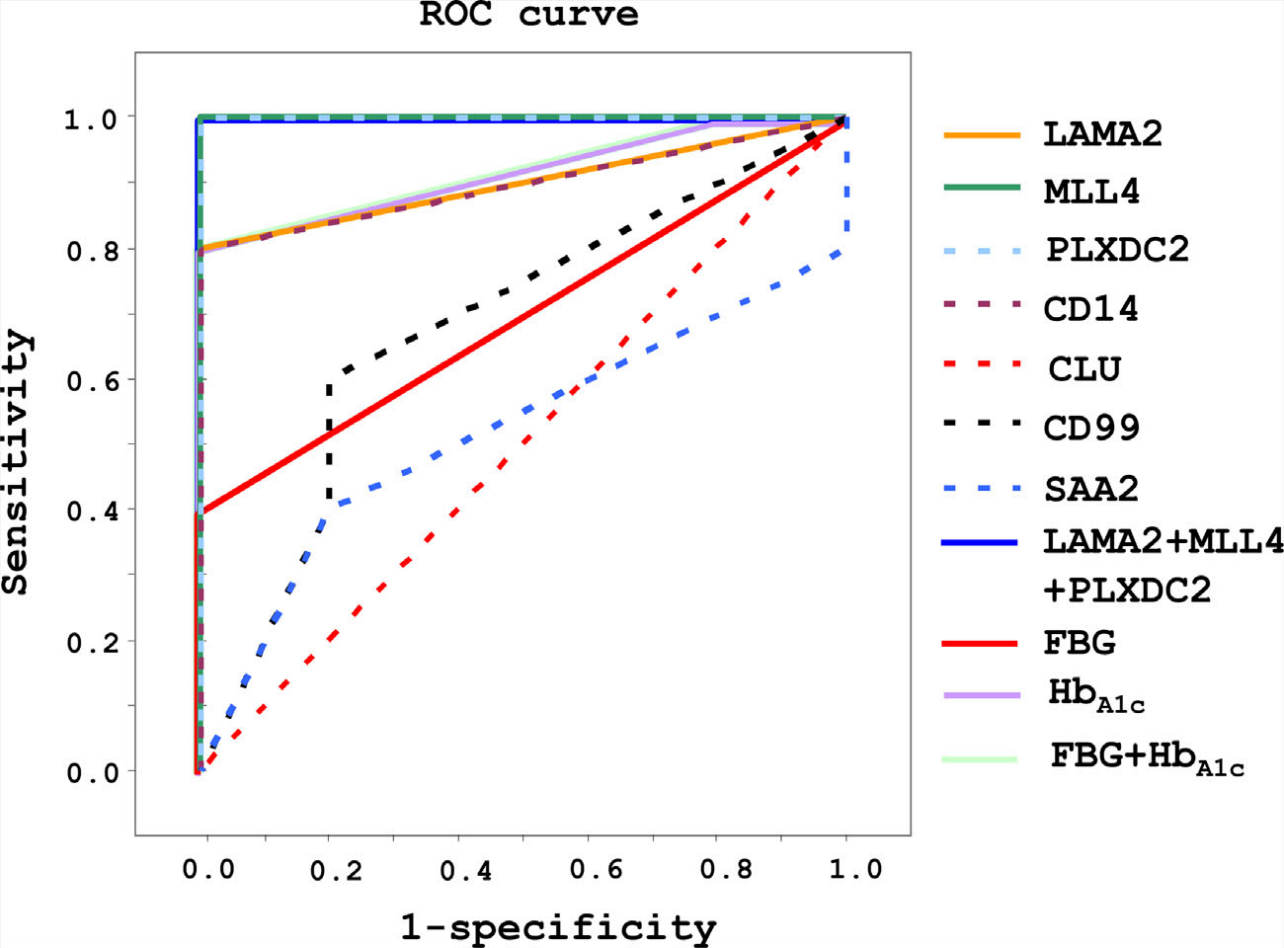
Diagnostic efficacy and value of LAMA2, MLL4, PLXDC2, CD14, CLU, CD99 and SAA2 in healthy and pre-diabetic sera of human origin. After immunoblotting analysis, diagnosis efficacy was analyzed using a ROC curve. Sensitivity, specificity, and accuracy were evaluated for diagnostic value.

The ROC curve was used as a diagram to illustrate the diagnostic efficacy of the serum LAMA2, MLL4, PLXDC2, CD14, CLU, CD99 and SAA2 as their discrimination thresholds. The AUC of the ROC curves was used to evaluate the diagnostic value of each protein. The AUC of LAMA2, MLL4, PLXDC2, CD14, CLU, CD99, and SAA2 was 0.9, 1, 1, 0.9, 0.5, 0.7, and 0.5, respectively (**Figure 5** and **Table 1**). Compared to currently used markers, FBG and Hb_A1c_, LAMA2, MLL4 and PLXDC2, alone and in combination, had higher AUC than FBG, Hb_A1c_ and/or both (**Table 1**). The sensitivity, specificity and accuracy of LAMA2 were 80%, 100% and 100%, respectively (**Table 1**). The sensitivity, specificity, and accuracy were all 100% for both MLL4 and PLXDC2 (**Table 1**). In contrast, CLU, CD99 and SAA2 had the same sensitivity (100%), specificity (0%), and accuracy (50%) as CD14 (**Table 1**). Of note, LAMA2, MLL4 and PLXDC2 had better specificity and accuracy than the others (**Table 1**). Furthermore, the sensitivity, specificity, and accuracy of LAMA2, MLL4 and PLXDC2, alone and in combination, were better than FBG, Hb_A1c_ and both (**Table 1**). Taken together, the three novel markers alone and in combination had better diagnostic value than currently existing markers, FBG and Hb_A1c._ To further validate the clinical potential of the above novel biomarkers, we screened the level of LAMA2 in the sera of health volunteers (n=12) versus pre-diabetic patients (n=11). As a consequence, we found that patients with pre-diabetes had a 4 fold higher serum LAMA2 level than healthy subjects (**Table 2**).

**Table 1:** Diagnostic value of serum LAMA2, MLL4, PLXDC2, CD14, CLU, CD99 and SAA2, FBG and Hb_A1C_ level for pre-diabetes.

Biomarkers	AUC	Sensitivity	Specificity	Accuracy	*p* value
LAMA2	0.9	80%	100%	90%	<0.05
MLL4	1	100%	100%	100%	<0.01
PLXDC2	1	100%	100%	100%	<0.01
CD14	0.9	100%	0%	50%	<0.01
CLU	0.5	100%	0%	50%	>0.05
CD99	0.7	100%	0%	50%	>0.05
SAA2	0.5	100%	0%	50%	>0.05
LAMA2+MLL4 +PLXDC2	1	100%	100%	100%	–
FBG	0.7	40%	100%	70%	>0.05
Hb_A1C_	0.9	80%	80%	80%	<0.01
FBG+ Hb_A1C_	0.9	80%	80%	80%	–

Sensitivity, specificity, and accuracy of different serum markers for pre-diabetes were evaluated according to the immunoblotting analysis. The AUC of the ROC curve was obtained according to sensitivity and specificity. A p value of less than 0.05 was considered statistically significant.

**Table 2:** Characteristics of humans used in LAMA2 ELISA.

Clinical characteristics	Healthy (n=12)	Pre-diabetic (n=11)
Age (year)	58.2 ± 1.2	56.7 ± 1.8
BMI	25.3 ± 0.8	24.5 ± 0.7
FBG (mg/dL)	94.6 ± 0.8	110.5 ± 2.5***
LAMA2 (ng/ml)	350.1 ± 51.6	1524.6 ± 257***

The parameters of healthy volunteers and pre-diabetic patients are indicated as mean ± standard error. The parameters with significant change (p ≤ 0.05) between the healthy and pre-diabetic subjects are indicated with asterisk(s).

Overall, the data suggest that a combination of iTRAQ and MS techniques is able to identify serum proteins as potential markers for (pre-)diabetes. LAMA2, MLL4 and PLXDC2 may be suitable diagnostic markers for (pre-)diabetes (**Table 1**). According to our results, among these proteins, MLL4 and PLXDC2 are the most promising potential markers for diagnosis because of their higher AUC (**Table 1**).

## Discussion

Mass spectrometry (MS) is an essential methodology in proteomics for basic research and applications. For instance, it can be used for identification of functional molecules and pathways for mechanistic study and for disease diagnosis (43). The iTRAQ labeling of multiple samples is used to measure the relative level of differential proteins simultaneously (43). Therefore, the iTRAQ-LC-MS/MS technique is widely used for quantitative proteomics because this technique saves time and has fewer experimental steps than conventional proteomics (43). A pool of three control serum samples was used to compare three individual sera of patients with pre-diabetes because this pooling could reduce control variability. However, a caveat of this pooling was neglect of variability of three individual control samples.

Up until the present, different molecules have been developed for diabetic diagnosis, including FBG, Hb_A1C_, fructosamine, glycated albumin (GA) and oral glucose tolerance test (OGTT) (44). Moreover, genetic markers have also been proposed for diabetic diagnosis (13). However, such markers are not suitable for the diagnosis of pre-diabetes or diagnosis prior to the onset of diabetes because their expression is not proportional to the progression of the disease. Furthermore, their specificity and sensitivity may not be satisfactory (13, 44). Hence, this work demonstrated the feasibility of identifying and using protein markers with high specificity and sensitivity for pre-diabetic diagnosis. That is to say, up- and down-regulation of the protein markers preceded the symptoms of diabetes. Here, we identified 7 candidate proteins, LAMA2, MLL4, PLXDC2, CD14, CLU, CD99 and SAA2 from 1074 human serum proteins using quantitative proteomics, statistics and pathway analyses. After immunoblotting analysis of healthy patients as well as patients with pre-diabetes and brain stroke, LAMA2, MLL4, and PLXDC2 emerged as potential (pre-)diabetic markers. The use of these markers has some advantage over traditional markers such as blood glucose and Hb_A1C_ (**Figures 4** and **5**). First, LAMA2, MLL4 and PLXDC2 are novel diagnostic markers for pre-diabetes. Second, the 3 serum proteins have an undetectable level in healthy subjects and largely increase in pre-diabetic subjects (**Figure 4**). Third, these markers have certain specificity for pre-diabetes. Fourth, they have higher sensitivity and accuracy than the known markers, CD99 and CLU. Moreover, their specificity is 0-20% higher than FBG and Hb_A1C_ and their sensitivity is 20-40% higher than FBG and Hb_A1C_ (44). Moreover, these 3 markers have great potential to detect pre-diabetes. In the future, a large number of clinical specimens from healthy and (pre-)diabetic subjects need to be verified for their clinical use alone and in combination with other markers. In addition, whether or not MLL4, LAMA2, and PLXDC2 can be used as therapeutic molecules needs to be addressed.

LAMA2, a basement membrane protein, plays an important role in muscle function (45). Deficiency of LAMA2 is associated with muscular dystrophy and demyelinating neuropathy (46). Moreover, abnormal skeletal muscle metabolic function causes insulin resistance in T2D patients (47). People in the late stage of diabetes have several diabetic complications including neuropathy, nephropathy, retinopathy and so on (48). Furthermore, it has been reported that transient neonatal diabetes mellitus (TNDM) is typically caused by imprinting aberrations in chromosomes located on LAMA2 (49). In our study, LAMA2 level in pre-diabetic patients was higher than in healthy subjects, implying that LAMA2 could be a promising marker in (pre-)diabetes and its complications. MLL4 plays a central role in transcription activation (50). Some evidence has shown that MLL4 is associated with cancer regulation (50). Furthermore, MLL4 was reported to bind to the MAFA and MAFB transcription factors to regulate islet β-cell function (24). The pre-diabetic patients had higher MLL4 level compared to healthy groups. Therefore, MLL4 might be a good candidate for (pre-)diabetic diagnosis. PLXDC2 is a transmembrane protein related to the developing central nervous system (51). It has been reported that the genetic variant near the PLXDC2 gene has an impact on the risk of primary open-angle glaucoma by increasing intraocular pressure in the Japanese population (52). Moreover, PLXDC2 level in the serum of pre-diabetic patients was higher than healthy groups. Thus, the results suggest that PLXDC2 may be a potential marker in (pre-)diabetes and its complications. Although CD14, CLU, CD99, and SAA2 have been reported to be protein markers for insulin resistance, obesity, and inflammation, our data showed that they only had a modest difference in expression level between healthy and pre-diabetic sera. Overall the data also illustrate the usefulness of the strategy of identifying human serum proteins as (pre-)diabetic markers. Although LAMA2 and CLU were commonly increased in pre-diabetic patients and mice, expression change of MLL4, PLXDC2, CD14, CD99, and SAA2 were found in human sera, but not in mouse counterparts (**Supplementary Tables 4** and **5**). Moreover, seven mouse serum proteins selected for biomarkers were not statistically significant because their FDR adjusted *p* values were over 0.05 (**Supplementary Table 5**). The data revealed that mouse serum proteins were less useful for human clinical prediction than human serum proteins. The data suggest the possible unreliability of using diabetic mouse models to seek the diagnostic markers for human (pre-)diabetes (**Figure 1**; **Supplementary Figure 1**; **Supplementary Table 4** and **Supplementary Table 5**) (41). Additionally, comparison of the expression level of LAMA2, MLL4, PLXDC2, CD14, CLU, CD99 and SAA2 in sera of healthy volunteers and patients with stroke and pre-diabetes suggests that LAMA2, MLL4 and PLXDC2 have better specificity for (pre-)diabetes than CD14, CLU, CD99 and SAA2 (**Figure 4** and **Supplementary Figure 3**) (41). LAMA2, MLL4 and PLXDC2 are specifically expressed in (pre-)diabetic sera; CD99 is expressed in the sera of both healthy and (pre-)diabetic patients; and CD14, CLU and SAA2 are expressed in the sera of healthy, (pre-)diabetic and stroke subjects (**Figure 4** and **Supplementary Figure 3**) (41). We did not test the 13 serum proteins, which were down-regulated in pre-diabetic patients for two reasons: (1) the 13 proteins are functionally irrelevant to diabetes; and (2) the relative expression levels of the 13 proteins (0.6- to 0.8-fold decrease) are lower than those of the top 7 serum proteins (1.3- to 2.7-fold increase).

Western blotting analysis and ELISA assays confirmed that LAMA2, MLL4 and/or PLXDC2 can be applied for (pre-)diabetic diagnosis (**Figure 4** and **Table 2**). In comparison with FBG and Hb_A1C_, LAMA2, MLL4 and PLXDC2 have higher sensitivity, specificity and accuracy than FBG (**Table 1**). MLL4 and PLXDC2 have higher sensitivity, specificity and accuracy than Hb_A1C_ (**Table 1**). However, Hb_A1C_ had lower sensitivity (35%) in pre-diabetes in a clinical trial (44). Therefore, the combination of these three novel proteins have potential for development of a novel and reliable method of (pre-)diabetic diagnosis. Furthermore, one advantage of using these (pre-)diabetic markers was that we could detect pre-diabetes in patients whose FBG (100-125 mg/dL) and Hb_A1c_ (5.7-6.5%) were relatively low and unstable compared to those during diabetes. The data support the superiority of our novel biomarkers in (pre-)diabetic diagnosis in comparison with the two traditional markers, FBG and Hb_A1c_. Moreover, LAMA2, MLL4 and PLXDC2 may be worth investigating for diabetes development.

## Data Availability Statement

The proteomics data is deposited to the ProteomeXchange Consortium *via* the PRIDE partner repository with the dataset identifier PXD017472.

## Ethics Statement

The studies involving human participants were reviewed and approved by the IRB/REC of the China Medical University hospital (IRB No. CMUH105REC2001). The patients/participants provided their written informed consent to participate in this study. The animal study was reviewed and approved by the protocol of the Institutional Animal Care and Use Committee of Academia Sinica (Protocol no. 12-12-478). Written informed consent was obtained from the individual(s) for the publication of any potentially identifiable images or data included in this article.

## Author Contributions

M-TY, T-FK, M-YS, Y-CC, and B-YL performed and analyzed the experiments. W-HC and Y-RC conducted the mass spectroscopy analysis. W-CY conceived the project idea, supervised the project, and wrote the manuscript. C-WY and Y-JT performed statistical analysis of the data. All authors contributed to the article and approved the submitted version.

## Funding

This work was supported by the Ministry of Science and Technology of Taiwan (MOST 108-3111-Y-001-057 and MOST 108-2823-8-001-003). 

## Conflict of Interest

The authors declare that the research was conducted in the absence of any commercial or financial relationships that could be construed as a potential conflict of interest.

## References

[1] Cerf ME. Beta Cell Dysfunction and Insulin Resistance. Front Endocrinol (2013) 4:37. 10.3389/fendo.2013.00037 PMC3608918 23542897

[2] Diabetes Atlas SEC. Idf DIABETES Atlas: International Diabetes Federation. Belgium: International Diabetes Federation Press (2015).

[3] Berndt P Evers S Foser S Fountoulakis M Martin ML Sebokova E. Cd99 as Target/Marker for Insulin Resistance. International Patent WO2006063733A1. Geneva, Switzerland: World Intellectual Property Organization (2006).

[4] Carlsson LM Peltonen M Ahlin S Anveden A Bouchard C Carlsson B. Bariatric Surgery and Prevention of Type 2 Diabetes in Swedish Obese Subjects. N Engl J Med (2012) 367(8):695–704. 10.1056/NEJMoa1112082 22913680

[5] Denham J O’Brien BJ Charchar FJ. Telomere Length Maintenance and Cardio-Metabolic Disease Prevention Through Exercise Training. Sports Med (2016) 46(9):1213–37. 10.1007/s40279-016-0482-4 26914269

[6] Tuomilehto J Lindstrom J Eriksson JG Valle TT Hamalainen H Ilanne-Parikka P. Prevention of Type 2 Diabetes Mellitus by Changes in Lifestyle Among Subjects With Impaired Glucose Tolerance. New Engl J Med (2001) 344(18):1343–50. 10.1056/Nejm200105033441801 11333990

[7] Pan XR Li GW Hu YH Wang JX Yang WY An ZX. Effects of Diet and Exercise in Preventing NIDDM in People With Impaired Glucose Tolerance. The Da Qing IGT and Diabetes Study. Diabetes Care (1997) 20(4):537–44. 10.2337/diacare.20.4.537 9096977

[8] Horikawa Y Yamasaki T Nakajima H Shingu R Yoshiuchi I Miyagawa J. Identification of a Novel Variant in the Phosphoenolpyruvate Carboxykinase Gene Promoter in Japanese Patients With Type 2 Diabetes. Horm Metab Res (2003) 35(5):308–12. 10.1055/s-2003-41307 12916001

[9] Vendrell J Fernandez-Real JM Gutierrez C Zamora A Simon I Bardaji A. A Polymorphism in the Promoter of the Tumor Necrosis Factor-Alpha Gene (-308) is Associated With Coronary Heart Disease in Type 2 Diabetic Patients. Atherosclerosis (2003) 167(2):257–64. 10.1016/s0021-9150(02)00429-x 12818408

[10] Metz TO Qian WJ Jacobs JM Polpitiya AD David GCII Smith RD. Serum Markers for Type II Diabetes Mellitus. United States patent US8673644B2. Virginia, U.S.: United States Patent and Trademark Office (2014).

[11] Rao AA Sridhar GR Srinivas B Das UN. Bioinformatics Analysis of Functional Protein Sequences Reveals a Role for Brain-Derived Neurotrophic Factor in Obesity and Type 2 Diabetes Mellitus. Mel Hypotheses (2008) 70(2):424–9. 10.1016/j.mehy.2007.03.034 17553627

[12] Gomez-Cardona EE Hernandez-Dominguez EE Velarde-Salcedo AJ. Alberto-Barrera-Pacheco, Diaz-Gois a, De Leon-Rodriguez A, Et al. 2D-DIGE as a Strategy to Identify Serum Biomarkers in Mexican Patients With Type-2 Diabetes With Different Body Mass Index. Sci Rep (2017) 7:46536. 10.1038/srep46536 28425473 PMC5397846

[13] Takahashi E Okumura A Unoki-Kubota H Hirano H Kasuga M Kaburagi Y. Differential Proteome Analysis of Serum Proteins Associated With the Development of Type 2 Diabetes Mellitus in the KK-A(y) Mouse Model Using the iTRAQ Technique. J Proteomics (2013) 84:40–51. 10.1016/j.jprot.2013.03.014 23545169

[14] Tsalamandris S Antonopoulos AS Oikonomou E Papamikroulis GA Vogiatzi G Papaioannou S. The Role of Inflammation in Diabetes: Current Concepts and Future Perspectives. Eur Cardiol (2019) 14(1):50–9. 10.15420/ecr.2018.33.1 PMC6523054 31131037

[15] Fernandez-Real JM Perez del Pulgar S Luche E Moreno-Navarrete JM Waget A Serino M. CD14 Modulates Inflammation-Driven Insulin Resistance. Diabetes (2011) 60(8):2179–86. 10.2337/db10-1210 PMC3142089 21700881

[16] Fogelstrand L Hulthe J Hulten LM Wiklund O Fagerberg B. Monocytic Expression of CD14 and CD18, Circulating Adhesion Molecules and Inflammatory Markers in Women With Diabetes Mellitus and Impaired Glucose Tolerance. Diabetologia (2004) 47(11):1948–52. 10.1007/s00125-004-1553-x 15558232

[17] Scheja L Heese B Zitzer H Michael MD Siesky AM Pospisil H. Acute-Phase Serum Amyloid A as a Marker of Insulin Resistance in Mice. Exp Diabetes Res (2008) 2008:230837. 10.1155/2008/230837 18584041 PMC2435226

[18] Manara MC Pasello M Scotlandi K. CD99: A Cell Surface Protein With an Oncojanus Role in Tumors. Genes (2018) 9(3):159. 10.3390/genes9030159 PMC5867880 29534016

[19] Lee EJ Lee HG Park SH Choi EY Park SH. CD99 Type II is a Determining Factor for the Differentiation of Primitive Neuroectodermal Cells. Exp Mol Med (2003) 35(5):438–47. 10.1038/emm.2003.57 14646598

[20] Pasello M Manara MC Scotlandi K. CD99 At the Crossroads of Physiology and Pathology. J Cell Commun Signal (2018) 12(1):55–68. 10.1007/s12079-017-0445-z 29305692 PMC5842202

[21] Park S Mathis KW Lee IK. The Physiological Roles of Apolipoprotein J/clusterin in Metabolic and Cardiovascular Diseases. Rev Endocr Metab Disord (2014) 15(1):45–53. 10.1007/s11154-013-9275-3 24097125

[22] Peix L Evans IC Pearce DR Simpson JK Maher TM McAnulty RJ. Diverse Functions of Clusterin Promote and Protect Against the Development of Pulmonary Fibrosis. Sci Rep (2018) 8(1):1906. 10.1038/s41598-018-20316-1 29382921 PMC5789849

[23] Ling IF Bhongsatiern J Simpson JF Fardo DW Estus S. Genetics of Clusterin Isoform Expression and Alzheimer’s Disease Risk. PloS One (2012) 7(4):e33923. 10.1371/journal.pone.0033923 22506010 PMC3323613

[24] Scoville DW Cyphert HA Liao L Xu J Reynolds A Guo S. MLL3 and MLL4 Methyltransferases Bind to the MAFA and MAFB Transcription Factors to Regulate Islet Beta-Cell Function. Diabetes (2015) 64(11):3772–83. 10.2337/db15-0281 PMC4613979 26180087

[25] Liang Y Li G Chen S He R Zhou X Chen Y. Muscle MRI Findings in a One-Year-Old Girl With Merosin-Deficient Congenital Muscular Dystrophy Type 1A Due to LAMA2 Mutation: A Case Report. BioMed Rep (2017) 7(2):193–6. 10.3892/br.2017.935 PMC5526140 28804634

[26] Miller-Delaney SFC Lieberam I Murphy P Mitchell KJ. Plxdc2 is a Mitogen for Neural Progenitors. PloS One (2011) 6(1):e14565. 10.1371/journal.pone.0014565 21283688 PMC3024984

[27] Nelson SM Panagiotou OA Anic GM Mondul AM Mannisto S Weinstein SJ. Metabolomics Analysis of Serum 25-Hydroxy-Vitamin D in the Alpha-Tocopherol, Beta-Carotene Cancer Prevention (ATBC) Study. Int J Epidemiol (2016) 45(5):1458–68. 10.1093/ije/dyw148 PMC5100614 27524818

[28] Bahnson ES Kassam HA Moyer TJ Jiang W Morgan CE Vercammen JM. Targeted Nitric Oxide Delivery by Supramolecular Nanofibers for the Prevention of Restenosis After Arterial Injury. Antioxid Redox Signal (2016) 24(8):401–18. 10.1089/ars.2015.6363 PMC4782035 26593400

[29] Lapolla A Porcu S Traldi P. Some Views on Proteomics in Diabetes. Clin Chem Lab Med (2011) 49(6):943–57. 10.1515/Cclm.2011.151 21410413

[30] Sundsten T Ortsater H. Proteomics in Diabetes Research. Mol Cell Endocrinol (2009) 297(1-2):93–103. 10.1016/j.mce.2008.06.018 18657591

[31] Herder C Karakas M Koenig W. Biomarkers for the Prediction of Type 2 Diabetes and Cardiovascular Disease. Clin Pharmacol Ther (2011) 90(1):52–66. 10.1038/clpt.2011.93 21654741

[32] Nakatani S Kakehashi A Ishimura E Yamano S Mori K Wei M. Targeted Proteomics of Isolated Glomeruli From the Kidneys of Diabetic Rats: Sorbin and SH3 Domain Containing 2 is a Novel Protein Associated With Diabetic Nephropathy. Exp Diabetes Res (2011) 2011:979354. 10.1155/2011/979354 PMC3189611 22007191

[33] Mullen W Delles C Mischak H Action EC. Urinary Proteomics in the Assessment of Chronic Kidney Disease. Curr Opin Nephrol Hy (2011) 20(6):654–61. 10.1097/MNH.0b013e32834b7ffa 21885967

[34] Prunotto M Ghiggeri G Bruschi M Gabbiani G Lescuyer P Hocher B. Renal Fibrosis and Proteomics: Current Knowledge and Still Key Open Questions for Proteomic Investigation. J Proteomics (2011) 74(10):1855–70. 10.1016/j.jprot.2011.05.031 21642026

[35] Kim HJ Kim PK Yoo HS Kim CW. Comparison of Tear Proteins Between Healthy and Early Diabetic Retinopathy Patients. Clin Biochem (2012) 45(1-2):60–7. 10.1016/j.clinbiochem.2011.10.006 22040812

[36] Merchant ML Klein JB. Proteomic Discovery of Diabetic Nephropathy Biomarkers. Adv Chronic Kidney D (2010) 17(6):480–6. 10.1053/j.ackd.2010.09.001 PMC2987606 21044770

[37] VanGuilder HD Bixler GV Kutzler L Brucklacher RM Bronson SK Kimball SR. Multi-Modal Proteomic Analysis of Retinal Protein Expression Alterations in a Rat Model of Diabetic Retinopathy. PloS One (2011) 6(1):e16271. 10.1371/journal.pone.0016271 21249158 PMC3020973

[38] Kim K Kim SJ Yu HG Yu J Park KS Jang IJ. Verification of Biomarkers for Diabetic Retinopathy by Multiple Reaction Monitoring. J Proteome Res (2010) 9(2):689–99. 10.1021/pr901013d 20020744

[39] Pringels S Van Damme N De Craene B Pattyn P Ceelen W Peeters M. Clinical Procedure for Colon Carcinoma Tissue Sampling Directly Affects the Cancer Marker-Capacity of VEGF Family Members. BMC Cancer (2012) 12:515. 10.1186/1471-2407-12-515 23148666 PMC3534223

[40] Tong W Ye F He L Cui L Cui M Hu Y. Serum Biomarker Panels for Diagnosis of Gastric Cancer. Onco Targets Ther (2016) 9:2455–63. 10.2147/OTT.S86139 PMC4853138 27217769

[41] Yang MT Chang WH Kuo TF Shen MY Yang CW Tien YJ. Identification of Novel Biomarkers for Pre-Diabetic Diagnosis Using a Combinational Approach. Front Endocrinol (2021) 12:641336. 10.3389/fendo.2021.641336 PMC8113970 33995275

[42] Mei Q Chen X Xiang L Liu Y Su Y Gao Y. DNA Barcode for Identifying Folium Artemisiae Argyi From Counterfeits. Biol Pharm Bull (2016) 39(9):1531–7. 10.1248/bpb.b16-00336 27582332

[43] Trinh HV Grossmann J Gehrig P Roschitzki B Schlapbach R Greber UF. iTRAQ-based and Label-Free Proteomics Approaches for Studies of Human Adenovirus Infections. Int J Proteomics (2013) 2013:581862. 10.1155/2013/581862 23555056 PMC3608280

[44] Dorcely B Katz K Jagannathan R Chiang SS Oluwadare B Goldberg IJ. Novel Biomarkers for Prediabetes, Diabetes, and Associated Complications. Diabetes Metab Synd Ob (2017) 10:345–61. 10.2147/DMSO.S100074 PMC5565252 28860833

[45] Kuang W Xu H Vachon PH Liu L Loechel F Wewer UM. Merosin-Deficient Congenital Muscular Dystrophy. Partial Genetic Correction in Two Mouse Models. J Clin Invest (1998) 102(4):844–52. 10.1172/JCI3705 PMC508948 9710454

[46] O’Brien DP Johnson GC Liu LA Guo LT Engvall E Powell HC. Laminin Alpha 2 (Merosin)-Deficient Muscular Dystrophy and Demyelinating Neuropathy in Two Cats. J Neurol Sci (2001) 189(1-2):37–43. 10.1016/s0022-510x(01)00559-7 11535231

[47] Phielix E Mensink M. Type 2 Diabetes Mellitus and Skeletal Muscle Metabolic Function. Physiol Behav (2008) 94(2):252–8. 10.1016/j.physbeh.2008.01.020 18342897

[48] Cade WT. Diabetes-Related Microvascular and Macrovascular Diseases in the Physical Therapy Setting. Phys Ther (2008) 88(11):1322–35. 10.2522/ptj.20080008 PMC2579903 18801863

[49] Andrade RC Nevado J de Faria Domingues de Lima MA Saad T Moraes L Chimelli L. Segmental Uniparental Isodisomy of Chromosome 6 Causing Transient Diabetes Mellitus and Merosin-Deficient Congenital Muscular Dystrophy. Am J Med Genet A (2014) 164A(11):2908–13. 10.1002/ajmg.a.36716 25124546

[50] Sze CC Shilatifard A. MLL3/MLL4/COMPASS Family on Epigenetic Regulation of Enhancer Function and Cancer. Cold Spring Harb Perspect Med (2016) 6(11):a026427. 10.1101/cshperspect.a026427 27638352 PMC5088509

[51] Miller SF Summerhurst K Runker AE Kerjan G Friedel RH Chedotal A. Expression of Plxdc2/TEM7R in the Developing Nervous System of the Mouse. Gene Expr Patterns (2007) 7(5):635–44. 10.1016/j.modgep.2006.12.002 17280871

[52] Mabuchi F Mabuchi N Takamoto M Sakurada Y Yoneyama S Kashiwagi K. Genetic Variant Near PLXDC2 Influences the Risk of Primary Open-Angle Glaucoma by Increasing Intraocular Pressure in the Japanese Population. J Glaucoma (2017) 26(11):963–6. 10.1097/IJG.0000000000000790 28930887

