# Supplementary material for: Identification of Novel Biomarkers for Pre-diabetic Diagnosis Using a Combinational Approach
Source: Front Endocrinol (Lausanne). 2021 Apr 28;12:641336. doi: 10.3389/fendo.2021.641336 (PMC8113970; doi:10.3389/fendo.2021.641336)
Supplement: Supplementary file 1 [file DataSheet_1.doc]

**Research article**

**Identification of novel biomarkers for pre-diabetic diagnosis using a combinational approach**

Meng-Ting Yang^1^, Wei-Hung Chang^1^, Tien-Fen Kuo^1^, Ming-Yi Shen^2^, Chu-Wen Yang^3^, Yin-Jing Tien^4^, Bun-Yueh Lai^1^, Yet-Ran Chen^1^, Yi-Cheng Chang^5^, and Wen-Chin Yang^1,6,7,8,9*^

^1^Agricultural Biotechnology Research Center, Academia Sinica, Taipei, Taiwan.

^2^Graduate Institute of Clinical Medical Science, China Medical University Taichung, Taiwan.

^3^Department of Microbiology, Soochow University, Taipei, Taiwan.

^4^Institute for Information Industry, Taipei, Taiwan.

^5^Graduate Institute of Medical Genomics and Proteomics, National Taiwan University, Taipei, Taiwan.

^6^Department of Institute of Biotechnology, National Taiwan University, Taipei, Taiwan.

^7^Institute of Pharmacology, National Yang-Ming University, Taipei, Taiwan.

^8^Department of Aquaculture, National Taiwan Ocean University, Keelung, Taiwan.

^9^Biotechnology Center, National Chung Hsing University, Taichung, Taiwan.

*Corresponding author:

Wen-Chin Yang, Agricultural Biotechnology Research Center, Academia Sinica, Taiwan. Tel.: 886-2-27872076; Fax: 886-2-27822245; and Email: wcyang@gate.sinica.edu.tw

Keywords: marker, proteomics, serum protein, type 2 diabetes, diagnosis

**Supplementary information**

**Materials and Methods**

**Mice serum samples collection.** C57Bl/6J and C57Bl/6J obese (db/db) mice were obtained from the National Laboratory Animal Center (Taipei, Taiwan) and the Jackson Laboratory (Bar Harbor, ME, USA), respectively. At 4 and 6 weeks of age, body weights, FBG, Hb_A1C_, TRIG, total cholesteral, high-density lipoprotein cholesterol, LDL, insulin and albumin level were measured as previously mentioned (n = 3/each group). Blood samples were obtained from the mice which had fasted for 16 hours. The serum was separated from whole blood by centrifugation and stored at -80°C. The mice were housed and fed standard mouse chow and water in a specific pathogen-free animal room with controlled temperature (22 ± 2^o^C), humidity (55 ± 10%) and light/dark cycle (12 hours/12 hours). All the animals were cared for based on the protocol of the Institutional Animal Care and Use Committee of Academia Sinica (Protocol no. 12-12-478).

**Serum sample processing and data analysis.** In order to decrease the sample complexity for the detection and identification of low-abundance proteins, depletion of abundant proteins, protein digestion, iTRAQ labeling and strong cation exchange chromatography fractionation were conducted. Then, the samples were analyzed by LC-MS/MS. Relative protein ratio and peptide identification were calculated using a database. Protein signaling pathways, functional and immunoblotting analysis were analyzed. The details of processing and data analysis are reported in the main text of manuscript.

**Statistical analysis.** The data are expressed as mean ± standard deviation of the mean. Student’s *t*-test was used to compare the difference between healthy volunteers and patients. A *p* value less than 0.05 was considered statistically significant. False discovery rate (FDR) was used to adjust proteomic *p* value for multiple comparisons.

**Supplementary Figures**

Health (n=3)

Pre-diabetes (n=3)

**
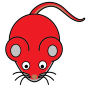

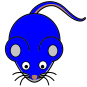

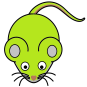

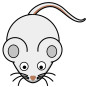

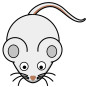

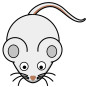
**

Labeled peptide pooling

SCX chromatography

Mascot analysis

LC-MS/MS

Protein labeling by iTRAQ

114m

115m

116m

117m

995 serum proteins

2 (LAMA2, CLU) of serum proteins which were the same as the potential markers from humans were identified

Identification of the proteins same in both humans and mice

**Supplementary Figure 1. Flow chart indicating the experimental designs for discovery of mouse serum proteins from healthy and pre-diabetic mice.** Serum samples were collected from 3 healthy mice and 3 pre-diabetic mice after 16 hours of fasting. Serum from 3 heathy mice were pooled together and labeled with iTRAQ 114m. Serum from 3 pre-diabetic mice was labeled with iTRAQ 115m, 116m and 117m respectively. Subsequently, four of them were pooled together for iTRAQ-based discovery. Finally, the mice serum proteins that were the same as the human serum protiens were identified.

a. Biological process


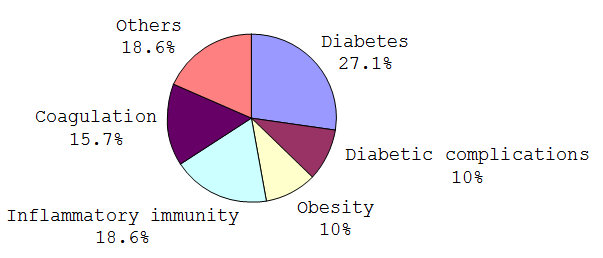


b. Molecular functions


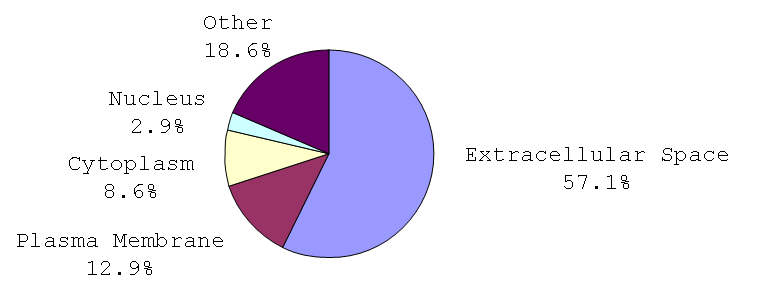


c. Cellular components


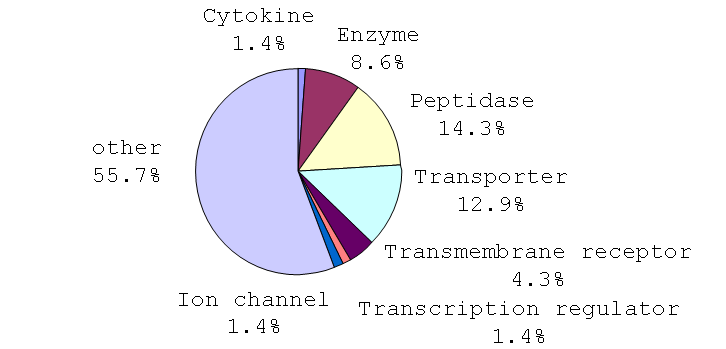


**Supplementary Figure 2. Functional analysis of 70 human serum proteins.** Gene ontology for biological process (a), molecular functions (b) and cellular components (c) of the selecvtive serum proteins with statistical significance (*p* < 0.05) in pre-diabetic patients compared to healthy volunteers.

**
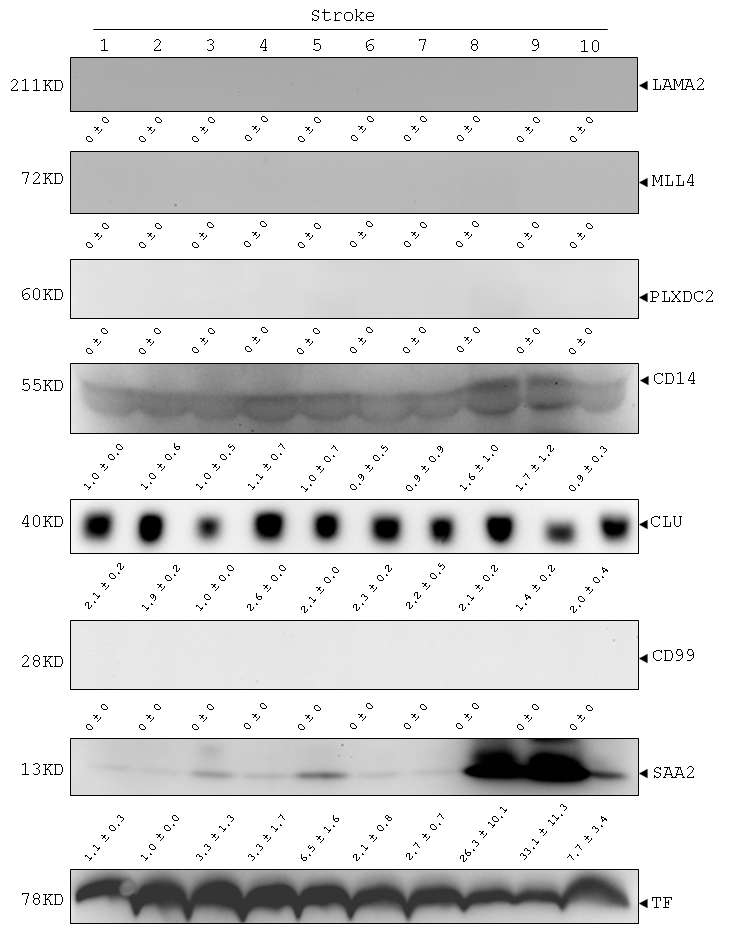
**

**Supplmentary Figure 3. Immunoblotting analysis of LAMA2, MLL4, PLXDC2, CD14, CLU, CD99 and SAA2 in stroke sera of human origin.** Serum samples of the stroke subjects were collected and then lysed with lysis buffer. After centrifugation, total lysates were prepared for immunoblotting analysis with the antibodies as indicated.

**Supplementary Tables**

**Supplementary Table 1.** Characteristics of humans.

| Clinical characteristics | Healthy  (n=3) | Pre-diabetic (n=3) |
| --- | --- | --- |
| Age (year) | 26.7 ± 0.9 | 60.7 ± 5.8** |
| BMI | 23.9 ± 2.9 | 23.4 ± 1.3 |
| FBG (mg/dL) | 86.7 ± 0.9 | 112 ± 3.2** |
| Hb_A1c_ (%) | 5.3 ± 0.2 | 6.5 ± 0.2* |
| TRIG (mg/dL) | 79 ± 10.4 | 102 ± 18.6 |
| TC (mg/dL) | 169.3 ± 20.2 | 161.3 ± 2.8 |
| HDL (mg/dL) | 57.5 ± 7.4 | 50 ± 5.9 |
| LDL (mg/dL) | 79 ± 11 | 91.3 ± 8.4 |
| Fasting insulin (uU/L) | 3.5 ± 0.1 | 10.5 ± 1.6* |
| Albumin (g/dL) | 5.2 ± 0.2 | 4.4 ± 0.1* |

The parameters of the groups are indicated as mean ± standard error. The parameters with significant change (*p* ≤ 0.05) between the healthy and pre-diabetic subjects are indicated with asterisk(s).

**Supplementary Table 2.** Characteristics of mice.

| Clinical characteristics | Healthy  (n=3) | Pre-diabetic (n=3) |
| --- | --- | --- |
| Age (week) | 4 | 6 |
| Body weight (g) | 16.1 ± 0.4 | 30.7 ± 0.7*** |
| FBG (mg/dL) | 80 ± 2.5 | 117.3 ± 2*** |
| Hb_A1c_ (%) | 3.7 ± 0.3 | 6.7 *** |
| TRIG (mg/dL) | 79.3 ± 4.3 | 118.3 ± 0.3*** |
| TC (mg/dL) | 119 ± 0.7 | 116 ± 5.5 |
| HDL (mg/dL) | 100.7 ± 0.7 | 104.7 ± 4.8 |
| LDL (mg/dL) | 15.9 ± 0.2 | 18*** |
| Fasting insulin (ng/ml) | 3.2 | 6 ± 0.1*** |
| Albumin (g/dL) | 3.1 ± 0.1 | 3.9 ± 0.3 |

The parameters of the groups are indicated as mean ± standard error. The parameters with significant change (*p* ≤ 0.05) between the healthy and pre-diabetic mice are indicated with asterisk(s).

**Supplementary Table 3.** Characteristics of humans for Western blots.

| Clinical characteristics | Healthy  (n=5) | Pre-diabetic (n=5) |
| --- | --- | --- |
| Age (year) | 28.6 ± 3.2 | 41.6 ± 6.4 |
| BMI | 21.7 ± 1.5 | 27.3 ± 1.4* |
| FBG (mg/dL) | 91.6 ± 2.6 | 94.2 ± 5.7 |
| Hb_A1c_ (%) | 5.4 ± 0.1 | 5.9 ± 0.1*** |

The parameters of the groups are indicated as mean ± standard error. The parameters with significant change (*p* ≤ 0.05) between the healthy and pre-diabetic subjects are indicated with asterisk(s).

**Supplementary Table 4. Seven huamn serum proteins selected for biomarkers.**

| SN | Protein name | Accession number | Average ratio | Mean log2 (ratio) | Functional categorization | FDR adjusted *p* value* |
| --- | --- | --- | --- | --- | --- | --- |
| 2 | LAMA2 | IPI00218725 | 2.27 | 1.43 | Diabetes | 0.04 |
| 14 | SAA2/4 | IPI00975939 | 1.82 | 0.86 | Diabetes | 0.04 |
| 16 | PLXDC2 | IPI00044369 | 1.74 | 0.80 | Diabetic complications | 0.04 |
| 23 | CD14 | IPI00029260 | 1.49 | 0.58 | Obesity | 0.04 |
| 32 | CD99 | IPI00253036 | 1.36 | 0.45 | Diabetes | 0.04 |
| 34 | MLL4 | IPI00218823 | 1.36 | 0.44 | Diabetes | 0.04 |
| 41 | CLU | IPI00291262 | 1.30 | 0.38 | Diabetes | 0.04 |

The proteins were selected for protin candidates based on the following criteria; *p* value < 0.01 and functions associated to diabetes, diabetic complications and obesity.

The asterisk (*) indicates false discovery rate (FDR) adjusted proteomic *p* value.

**Supplementary Table 5. Seven mouse serum proteins selected for biomarkers.**

| SN | Protein name | Accession number | Average ratio | Mean log2 (ratio) | Functional categorization | FDR adjusted *p* value* |
| --- | --- | --- | --- | --- | --- | --- |
| **1** | **LAMA2** | **IPI00309999** | **2.71** | **1.44** | **Diabetes** | **0.15** |
| 2 | GPX3 | IPI00133536 | 1.45 | 0.54 | Diabetes | 0.22 |
| 3 | A2M | IPI00454052 | 1.42 | 0.51 | Cirrhosis, nephrotic syndrome | 0.33 |
| 4 | APOH | IPI00322463 | 1.36 | 1.44 | Cancer | 0.45 |
| **5** | **CLU** | **IPI00320420** | **1.31** | **0.39** | **Diabetes** | **0.06** |
| 6 | F10 | IPI00331551 | 1.30 | 0.38 | Blood coagulation | 0.27 |
| 7 | TTR | IPI00127560 | 1.12 | 0.16 | Transporter of thyroxine | 0.38 |

The proteins were selected for protin candidates based on the following criteria; Mascot score > 20, unique peptide matches ≥ 1 and conserved between mouse sera (305 proteins) and human sera (70 proteins). The asterisk (*) indicates false discovery rate (FDR) adjusted proteomic *p* value.
